# Patient Perceptions of Text Messaging to Improve Antiretroviral Therapy Adherence: A Qualitative Study of Patients in a Ryan White Clinic

**DOI:** 10.1177/2325958218759209

**Published:** 2018-02-23

**Authors:** Elizabeth Sherman, Kevin Alan Clauson, Shara Elrod, Paula Eckardt, Fadi Alkhateeb, Robin Jacobs

**Affiliations:** 1 Nova Southeastern University College of Pharmacy, Fort Lauderdale, FL, USA; 2 Lipscomb University College of Pharmacy, Nashville, TN, USA; 3 University of North Texas System College of Pharmacy, Fort Worth, TX, USA; 4 Division of Infectious Diseases, Memorial Physician Group, Hollywood, FL, USA; 5 University of Texas at Tyler College of Pharmacy, Tyler, TX, USA; 6 Baylor College of Medicine, Houston, TX, USA

**Keywords:** HIV, SMS, text message, mobile health, qualitative

## Abstract

Evidence on the use of short message service (SMS) to improve medication adherence in people living with HIV (PLHIV) is mounting, yet qualitative research on patient perceptions regarding SMS content and utility for HIV/AIDS remains nascent. To explore the experience of receiving medication reminders via SMS among PLHIV, 45 uninsured and underinsured PLHIV nested within the intervention arm of a larger study received daily, 1-way SMS adherence reminders. Qualitative data were collected by face-to-face, structured interview and were analyzed using conventional content analysis methods. Three main themes emerged from the data: (1) reminders helping with adherence, (2) concerns about delivery modes, and (3) the need for confidentiality. Study findings offer enhanced focus on an emerging strategy in patient-centered HIV care: Equipped with greater context on the experiences of PLHIV using SMS adherence reminders, health-care providers can offer more targeted support and thereby maximize the benefits of this popular and powerful technology.

## Introduction

Medication nonadherence to antiretroviral therapy (ART) remains one of the most accessible, modifiable risk factors for disease progression in people living with HIV (PLHIV).^1,2^ However, as 2020 approaches, the United Nations goal of “90 90 90” (ie, by the year 2020, 90% of PLHIV know their HIV status, 90% of persons with diagnosed HIV receive sustained ART, and 90% of PLHIV receiving ART achieve virologic suppression)^3^ remains a daunting challenge. Although at a national level a growing number of countries are either on track to achieve the 90-90-90 target or have approached, met, or exceeded one or more components of the target, the countries lagging behind are in need of best practices across the treatment cascade.^3^ The use of mobile health (mHealth) tools, including the use of short message service (SMS; ie, text messaging), represents a low-cost method to leverage technology for improving medication adherence to ART^4,5^ inclusive of resource-limited settings.^6,7^ Reflecting the potential of this mHealth intervention, the most recent International Association of Providers of AIDS Care guidelines for optimizing the HIV care continuum include specific recommendations for using SMS to improve medication and appointment adherence.^8^

Although an increasing body of evidence on SMS to improve medication adherence in PLHIV is being generated,^9–11^ qualitative research on patient perceptions regarding SMS content and utility for HIV/AIDS treatment remains relatively underdeveloped.^12,13^ Accordingly, the current research was developed to assess patient satisfaction with ART medication reminders delivered via SMS in the context of an interventional study to improve medication adherence for uninsured and underinsured PLHIV in the United States. The purpose of the described study was to characterize patient perceptions regarding receipt, content, and utility of the SMS. A qualitative analysis approach was employed to evaluate participant feedback and to frame the feedback collectively into instructive themes for a broader understanding of the phenomenon of study.

## Methods

As part of a larger study evaluating the benefits of SMS medication adherence reminders on ART medication adherence, we recruited patients based on clinician referral from an HIV specialty primary care clinic receiving federal Ryan White funding to serve the uninsured and underinsured populations of South Florida. Participants met the following criteria: 18 years of age or greater, documented HIV infection, English literate, own a cell phone with SMS capability, HIV treatment naive, and initiating ART for the first time or started ART within 1 month of eligibility screening. Patients with dementia, blindness or severely impaired vision uncorrectable with eyeglasses, deafness or hearing problems uncorrectable with a hearing aid, or the inability to provide informed consent were excluded. Participants provided written informed consent prior to beginning the study. The institutional review boards of Nova Southeastern University, Memorial Healthcare System, and Florida Department of Health approved this research.

Following enrollment, participants were randomized by computer into 1 of 2 groups: SMS (intervention) group or no SMS (control) group. Data points were collected at 3 additional time points over the course of this 6-month study at the following intervals: 2 to 8 weeks, month 3, and month 6. Participants were given a US$20 gift card at the 2- to 8-week time point, a US$40 gift card at month 3 visit, and a US$60 gift card at month 6 visit as a participation incentive. Here, we report the qualitative findings from the SMS group, which were collected at month 3 (study midpoint) and month 6 (study conclusion).

Participants randomized to the SMS group received daily, automated 1-way ART reminders (ie, “Here’s to your health!”) at the time of their choosing to their mobile phone delivered via SMS (MEMOTEXT, Toronto, Ontario, Canada). Messages were delivered for a period of 6 months and, due to requirements of the text messaging company, messages were sent from a different phone number every day. A face-to-face 20-minute interview conducted by study investigators at month 3 and month 6 of the study consisted of 3 open-ended items to gather qualitative data on participants’ perceptions regarding using the SMS: (1) What do you like about receiving text message reminders? (2) What do you dislike about receiving text message reminders? (3) What would you change about receiving text message reminders? All interviews were conducted in a private space at the local community clinic. For analysis, study investigators transcribed participant responses and then reread their notes to the participants to ensure accuracy. Grammatical variations were retained to reflect the participants’ voices.

### Analytic Methods

Qualitative data collected from the open-ended items were analyzed using narrative frameworks to explore the language and context of the responses.^14–16^ By examining each participant’s viewpoint, the study illuminated the larger framework of the phenomenon of interest (ie, attitudes on SMS medication reminders). The approach to coding and analysis highlighted the patterns, themes, and categories of analysis emerging from the data. The data were coded for words and phrases to determine frequency. Moreover, concepts were coded exactly as they appeared, as well as if they appeared in different forms. For example, “confidential” might also appear as “very private” or “very discreet,” as the words and phrases were deemed similar enough that they could be coded as the same thing (ie, “privacy words”). Determining the level of implication allowed for coding not only for the word “private” but also for words that imply “confidentiality.” Irrelevant information was ignored, such as words “and” and “the” as they appear by themselves.

After creating the themes, successive qualitative analyses were conducted. A descriptive analysis developed an understanding of participants’ messages (ie, why they liked or disliked the text messages or things they would change about them). A thematic analysis elaborated structures of basic study constructs identified a priori and new constructs arising in the early phases of analysis. Test references were assembled and compared to a concept and determined to what extent respondents affirmed only single aspects or multiple aspects of a concept. Finally, a comparative analysis clarified differences in likes and dislikes about the text messages among participants and between month 3 and month 6.

## Results

Forty-five patients participated in the SMS arm of the study with a mean age of 37.5 years (Table 1). Participants were more likely to be male (62%), of black/African American race (71%), and heterosexual (64%). Forty-two percent of participants had a baseline HIV RNA log10 equal to or greater than 5.00 (42%), and the majority (53%) were prescribed a non-nucleoside reverse transcriptase inhibitor–based ART regimen. At entry into the study, 49% of participants sent and received fewer than 10 text messages per day.

**Table 1.** Study Participants’ Baseline Characteristics.^a^

Variable	Mean (Standard Deviation) or Frequency Count (%)
Age, years	37.5 (11.6)
Gender	
Male	28 (62%)
Female	17 (38%)
Race/ethnicity	
Black/African American	32 (71%)
Hispanic/Latino	9 (20%)
White, non-Hispanic	4 (9%)
HIV risk factor at screening	
Heterosexual sex	29 (64%)
Men having sex with men (MSM)	12 (27%)
MSM and heterosexual sex	2 (4%)
Unknown	2 (4%)
Average number of text messages sent and received each day	
0-9	22 (49%)
10-49	13 (29%)
50-99	4 (9%)
100+	6 (13%)
Antiretroviral regimen prescribed	
Nonnucleoside reverse transcriptase inhibitor	24 (53%)
Protease inhibitor	11 (25%)
Integrase strand transfer inhibitor	10 (22%)
HIV RNA log10	
2.00-3.99	12 (27%)
4.00-4.99	14 (31%)
5.00-5.99	15 (33%)
6.00+	4 (9%)

^a^N = 45.

From the data analysis, 3 main themes emerged: (1) reminders helping with adherence, (2) concerns about delivery modes, and (3) the need for confidentiality. There were no major differences in response themes between month 3 and month 6.

### Reminders Helping With Adherence

A major theme that emerged was the importance and usefulness of the SMS reminders. Participants appreciated the reminder and perceived that the messages helped them better adhere to the medication regimen, both at the midpoint and conclusion of the study. In this regard, 1 participant stated:

It’s a great idea and it’s really good. It’ll be 10:00 and my phone goes off and I don’t even have to look at it; I already know. I think it’s a great thing that you guys do it. [month 3]

Another participant similarly stated

I like it because it’s at 7:00 and I love that. If I leave work at night I leave at 7:00 so I can take my meds as I walk out the door. If I’m at home cooking dinner, it’s a reminder that it’s 7:00. I like it because it’s reminding me. [month 3]

Many participants expressed that the text messages helped them to remember to take their medications at times when they otherwise would have forgotten. One participant stated:

It’s a constant reminder to make sure I’m taking my meds. Without the text it would be difficult to remember to take them…I think they helped me to make taking my meds more routine. I won’t miss them. I can do it on my own, but without the messages it’s easy for me to say ‘Oh, I’ll just go to bed and not take it.’ When I get the text I stop to see who wrote me. Then I see it’s the reminder. So I take my meds then. [month 6]

Another participant concurred “[t]hey remind you because wherever I’m at if I hear it I stop whatever I’m doing and go get it. I know that I have to take it but believe it or not sometimes it just flips my mind. The text message gets me going. And I depend on it, too. If I don’t hear it I’m like it’s not time yet.” [month 6]

### Concerns About Delivery Modes

The second theme that emerged was related to logistical issues of the texting itself. Although many participants found the texts helpful in reminding them to take their medications, some felt they needed to have more control of the messages, such as having the capability to change the time of the text alert or even its content message.

I wish I could reply to the text message and say remind me in 30 minutes. Like reply 1 to resend in 30 minutes. Because sometimes I have cleared it out and was like oh. But I still remember when I get a text message from another person. Or if I could reply to change the timing, because I have to take my medication with food and even though I take it with dinner I’m not hungry at that time, so I wish I would change the time to 2 hours earlier when I ate. [month 3]

Some participants found the text messages somewhat confusing.

It’s a great reminder to not forget to take the medications. But sometimes you could be in situations where you get the text and “here’s to your health” is confusing and you forget what it’s for and you have to remind yourself what it means. I had to remember it was for the medications. [month 3]

Several participants mentioned they disliked that the text came from different phone numbers each time; however, others commented it was acceptable when they realized the message was a general message from the research study. One participant noted:

The messages from one phone number would be more assuring. [month 6]

Other text-related logistical issues identified included suggestions to have a 15- or 30-minute “snooze” option.

That I can’t snooze it. Or hit reply and say send me in 15 minutes because sometimes I’m not home and not by the pills. I might accidentally clear out the text message and usually I have it sitting there waiting. [month 6]

### The Need for Confidentiality

The third theme was related to confidentiality and privacy of the SMS intervention. Several participants mentioning privacy issues appreciated the confidential nature of the messages throughout the study.

I like how the text messages remind me to take my meds without revealing my condition at all. [month 3]

If somebody sees my message they don’t know what it’s for. [month 3]

I like it, it’s good. Only I know what the text means. If you get my phone you don’t know what that means. [month 6]

Overall, the participants were appreciative that the message, if by chance seen by others, would not disclose anything about their HIV status. They liked receiving reminders to take their medication that revealed no information on their health condition.

### Other Findings

#### Social support

While not a fully emerged theme per se, the need for social support was reported by several participants.

It’s a reminder that people care. [month 3]

I feel good about the texts. It says you remember me. [month 3]

I feel like someone’s taking care of me and my HIV problem. [month 6]

Over time, the SMS may have increased the patient’s sense that someone cared about them, but too few comments were made to make a generalization. Further research is needed to examine the effect of SMS adherence reminders on the social and emotional well-being of PLHIV and adherence.

#### Tailored content

A few participants mentioned the desire for more personalized text content to help them remember to take their medication.

I wish the message was something funny like “take your pills!!”. To me, “here’s to your health” sounds funny. If I could have a custom one, I would put “take your pills, [expletive]!” that would make me laugh every time.”[month 6]

I still think the message would be better if it said something like “hey don’t forget.” [month 6]

One participant suggested adding motivational content to the message.

Perhaps the message could be more motivational because the message is always the same. Maybe the message could change each day. [month 6]

## Discussion

The present study explores the experience of receiving daily SMS medication reminders among treatment-naive PLHIV in a federally funded US clinic serving uninsured and underinsured patients. Few studies have focused on this specific patient population. The study results illustrate that the experience with the SMS adherence intervention is characterized by reminders helping with adherence, concerns about delivery modes, and the need for confidentiality. Participants overall appreciated the utility and convenience of medication reminders delivered via an SMS mechanism.

Consistent with other research, study participants perceived that SMS reminders helped them improve their adherence to ART.^17^ Forgetfulness presents a barrier to medication adherence that can be addressed with SMS technology through regular reminders. However, participants also indicated their concerns about logistics of receiving messages, specifically how and when messages were delivered and the content of the messages. Participants in our study selected the time of day they wanted to receive the SMS at study enrollment and messages were sent at the same time every day. However, at follow-up, participants commented that this specific time might not be the most convenient. In another study examining patient satisfaction of SMS to improve medication adherence, patients indicated that messages received after the medication was to be consumed were not beneficial.^18^ In our study, investigators adjusted the time of the SMS based on patient preference at the month 3 visit. However, allowing patients the flexibility to easily change the time of SMS receipt themselves, as multiple participants suggested, offers a more patient-centered approach to care and may further improve outcomes. The apparent source of the SMS was a concern for some participants. Some indicated they preferred to receive the message from the same phone number. This corresponds with other research where participants also commented about the perceived source of the SMS.^19^ Short message service delivery modes are clearly a concern for some patients. Evidence-based research is therefore indicated to determine best practices. Participants also appreciated the confidential aspect of the message itself. Coding the message so that it did not contain the words “HIV” or “antiretrovirals” was pleasing to participants because it protected their health status from others.

A few participants perceived social support when receiving the SMS reminders. Similarly, a major theme described in several studies examining patient perceptions of SMS to improve medication adherence is the care and concern participants feel from their clinic or physician as a result of receiving the messages.^17,20,21^ The absence of a robust social support theme in our study may result from the relatively anonymous content of our message or from the lack of probing questions in our follow-up interviews.

Some participants felt the message content should be more tailored. Short message service personalization has been examined in an antihypertensive medication adherence study employing personalized messages such as “Happy Birthday” or messages signed by the patients’ provider.^21^ Participants reported a positive experience and described feeling “special” and “cared for.” Similarly, participants in an obesity treatment SMS study had positive attitudes about targeted or personalized messages.^22^ Although participants in our study reported SMS improved perceived medication adherence, adding more personalized SMS messages could further improve perceived social support and medication adherence.

Although the results of this qualitative study can add to knowledge regarding the experience of PLHIV, the results are not generalized to all HIV-infected adults. One of the main limitations of this study is that we utilized the experience of patients at a single federally funded clinic serving indigent and uninsured patients in the United States. Findings from this small sample cannot be extended or generalized to wider populations of PLHIV or to persons living in more rural settings. Additionally, because study investigators provided patient care as well as study-related interviews, participants may have felt inclined to provide professionally desirable responses. Also, notes were taken manually without a video or audio recorder. Even though the quotes were reread to the participants to ensure accuracy, there is always the possibility of human error. Finally, the majority of study participants received first-line HIV treatment with a nonnucleoside reverse transcriptase inhibitor, reflective of the study recruitment period during the years 2011 to 2014 and contemporaneous guideline-based treatment recommendations. This may limit the generalization of our results to contemporary regimens, including those that are largely integrase inhibitor based.

## Conclusion

The experience of adult PLHIV receiving daily SMS medication reminders is associated with reminders helping with adherence, concerns about delivery modes, and the need for confidentiality. Study findings offer enhanced focus on an emerging strategy in patient-centered HIV care: Equipped with greater context on the experiences of PLHIV using SMS adherence reminders, health-care providers can offer more targeted support and thereby maximize the benefits of this popular and powerful technology.

Although SMS is an acceptable means of providing support and motivation, it is important that health-care professionals learn about the experiences and specific needs of PLHIV to tailor the service to individual needs and to improve patient care. Prospective evaluations of larger scale SMS interventions are required to assess the resulting long-term behavior change in medication adherence.
